# Assessment of Non-invasive Markers for the Prediction of Esophageal Variceal Hemorrhage

**DOI:** 10.3389/fmed.2021.770836

**Published:** 2021-12-01

**Authors:** Shasha Li, Peng Huang, Andre J. Jeyarajan, Chao Ma, Ke Zhu, Chuanlong Zhu, Ning Jiang, Ming Li, Tuo Shao, Mingfeng Han, Lin Tan, Wenyu Lin

**Affiliations:** ^1^Department of Hepatology, The Second People's Hospital of Fuyang City, Fuyang, China; ^2^Department of Epidemiology, School of Public Health, Nanjing Medical University, Nanjing, China; ^3^Liver Center and Gastrointestinal Division, Department of Medicine, Massachusetts General Hospital, Harvard Medical School, Boston, MA, United States; ^4^Department of Gastroenterology, The Second People's Hospital of Fuyang City, Fuyang, China; ^5^Department of Infectious Disease, The First Affiliated Hospital of Nanjing Medical University, Nanjing, China; ^6^Department of Pneumology, The Second People's Hospital of Fuyang City, Fuyang, China

**Keywords:** esophageal varices (EVs), liver fibrosis, hemorrhage, Golgi protein 73 (GP73), population cohort study

## Abstract

**Background:** Esophageal variceal (EV) hemorrhage is a life-threatening consequence of portal hypertension in cirrhotic patients. Screening upper endoscopy and endoscopic variceal ligation to identify and treat EVs have contraindications, complications, and high costs. We sought to identify non-invasive tests (NITs) as alternatives to endoscopic EV screening.

**Methods:** In this case-control study, we retrospectively analyzed 286 cirrhotic patients treated for EVs at the Second People's Hospital of Fuyang City, China from January to December 2019. We applied ROC curve analysis to assess the accuracy of various NITs in predicting EV hemorrhage.

**Results:** There were significant differences between the hemorrhage and non-hemorrhage groups in median serum albumin (ALB) (*p* < 0.001), median bilirubin (TBIL) (*p* < 0.046), prothrombin (PT) time (*p* < 0.001), Golgi protein 73 (GP73; *p* = 0.012) and Child-Pugh (C-P) scores (*p* < 0.001). For ALB (cutoff <33.2g/L), PT time (cutoff > 14.2 seconds), GP73 (cutoff > 126.4 ng/ml), and C-P scores, the areas under the ROC curves (AUCs) were 73.4% (95% CI: 67.5–79.2), 68.6% (95% CI: 62.4–74.8), 62.2% (95% CI: 52.8–71.5) and 69.8% (95%CI: 63.8–75.8), respectively, with corresponding sensitives of 71.5, 59.8, 69.8, and 92.2% and specificities of 65.6%, 70.1%, 56.5%, and 38.6%. When ALB was combined with GP73, the AUC was 74.3% (95% CI: 66.1–82.5) with a sensitivity of 65.1% and specificity of 76.5%. When ALB, PT, and C-P scores were combined, the AUC was 76.5% (95% CI: 70.9–82.1) with a sensitivity of 79.5% and specificity of 64.3%. When ALB, PT, GP73, and C-P scores were combined, the AUC was 75.2% (95% CI: 67.3–83.1) with a sensitivity of 54.0% and specificity of 86.9%.

**Conclusion:** ALB, TBIL, GP73, and C-P scores, may be used to predict EV hemorrhage in cirrhotic patients. The combination of multiple NITs is better than a single index and can increase diagnostic performance.

## Introduction

Chronic liver disease can progress to cirrhosis, one of the leading causes of death worldwide. Cirrhosis-related complications account for approximately one million deaths each year, representing 3.5% of total global deaths annually (1). Esophageal varices (EVs) are enlarged abnormal veins in the distal esophagus that develop from elevated portal venous pressure due to increased resistance and portal blood flow (portal hypertension) in cirrhosis. Approximately 4% of cirrhotic patients with EVs suffer from variceal hemorrhage each year and the mortality rate within six weeks after bleeding is as high as 25% (2). Currently, there is no universal effective prophylactic treatment for EV hemorrhage (3). Non-selective beta blockers (NSBBs) are used to prevent bleeding in patients with EVs, but do not stop variceal growth (4, 5). Esophagogastroduodenoscopy (EGD) is used to identify and estimate the size of EVs and endoscopic variceal ligation is a prophylactic option for high-risk individuals. However, limitations of upper gastrointestinal (GI) endoscopy include its high costs and complications, including those associated with intravenous sedation (4, 6).

Transient elastography (TE) has been used to estimate liver stiffness (LS) and may serve as a new method to non-invasively diagnose portal hypertension (7, 8). The combination of three simple methods—LS, spleen size, and platelet count—into a single score (LSPS) has been shown to identify EVs with high accuracy in patients with compensated cirrhosis (9–11). According to the Baveno IV consensus, patients with a platelet count >150,000 and LS measurement <20kPa by TE can avoid screening upper endoscopy for EVs (12). However, it is important to identify simple, readily available non-invasive tests that can predict EV hemorrhage for those patients that do not satisfy the Baveno IV criteria, where TE or EGD is limited or not available, or the patient wishes to avoid endoscopy.

Several minimally- or non-invasive tests (NITs) have been proposed as alternatives to EGD for EV screening (4, 7). Golgi Phosphoprotein 2 (GOLPH2)/Golgi protein 73 (GP73) is one potential non-invasive measure, as it is a well-studied biomarker for liver fibrosis and hepatocellular carcinoma (HCC) (13). However, prior studies (14, 15) suggest that other NITs, such as the aspartate aminotransferase-to-platelet ratio index (APRI), aspartate-to-alanine aminotransferase ratio (AAR), fibrosis-4 index (FIB-4), fibrosis index (FI), King, Lok, Forns, and FibroIndex scores, have poor accuracy in diagnosing EVs (16–18). We therefore sought to analyze the accuracy of a combination of several different simple routinely available NITs, including FIB-4, spleen length, GP73, and other laboratory parameters, to determine their utility in predicting EV hemorrhage.

## Materials and Methods

### Patients and Study Design

In this case-control study, 286 patients with confirmed cirrhosis and esophageal varices (EVs) were recruited from January to December 2019 at the Second People's Hospital of Fuyang City. Cases included patients who experienced EV hemorrhage (EVH+) while controls were patients without hemorrhage (EVH-). All patients had endoscopically confirmed EVs, and diagnosis was in accordance with the standards established by the American Association for the Study of Liver Diseases (AASLD) (19). The inclusion criteria were as follows: (1) age 18–75 years, (2) presence of cirrhosis, and (3) gastroscopy that confirmed EVs. Patients with the following conditions were excluded: (1) liver cancer or other organ tumors, (2) refusal to undergo endoscopy, and (3) patients with large ascites.

### Laboratory Tests

Laboratory parameters obtained included alanine aminotransferase (ALT), aspartate aminotransferase (AST), gamma-glutamyl transferase (GGT), alkaline phosphatase (ALP), albumin (ALB), bilirubin (TBIL), prothrombin time (PT), International Normalized Ratio (INR), electrolytes, hemoglobin, hematocrit, and leukocyte and platelet (PLT) count. Complete blood count (CBC) was measured using the SYSMEX CA5100 automatic clotting analyzer (Siemens Healthcare, Erlangen, Germany) (20, 21). Liver function tests (LFTs) were assessed using the Hitachi 7600 fully automatic biochemical analyzer (20, 21). Serum GP73 was quantified using the UPT 3A-1800 Immunassay Analyzer.

Samples were collected when the patient was hemodynamically stable. For patients without EVH, laboratory tests recorded within two days before or after gastroscopy were included. Since hemorrhage can affect platelet count and other clinical parameters, we aimed to minimize this effect during sample collections. In patients with EVH, bloodwork was therefore performed before the bleeding event or two days after bleeding ceased.

### Patient Evaluation

The diagnosis of EVs was based on the 2008 Hangzhou consensus proposed by the Chinese Society of Gastroenterology, Chinese Society of Hepatology, and Chinese Society of Digestive Endoscopy. These criteria were derived from the AASLD practice guidelines, Baveno consensus, and Japanese Society for Portal Hypertension guidelines (12, 14, 22–24). Patients were listed as having no, mild, moderate, or severe varices as in previous reports (12, 14). Spleen size was routinely measured and reported on ultrasound examinations (Philips Ultrasound Machines) at The Second People's Hospital, Fuyang and other participation centers by experienced medical doctors. The formulas for the non-invasive scores utilized are reported below. The Child-Pugh score for Cirrhosis Mortality was also calculated (25–27).


(1) $$\begin{array}{l} APRI=AST\times ULN/platelet\left(10^{9}/L\right)\times \;100 \end{array}$$

(2) $$\begin{array}{l} FIB-4=Age\;\left(years\right)\times AST\;\left(U/L\right)/platelet\left(10^{9}/L\right)\\ \;\times \;\sqrt{ALT\left(U/L\right)} \end{array}$$

### Sample Size Calculation and Statistical Analysis

Data were analyzed using SPSS Statistics v25.0 (IBM Corp, USA). Continuous data are expressed as medians with interquartile range and categorical data as frequencies. Continuous variables were compared using the Mann-Whitney U test, while categorical data were compared with the Chi-squared test. NCSS-PASS software (v = 15.0) was used to estimate sample size. The parameters were as follows: α = 0.05 (two-side test), β = 0.1, N0 (case group) = N1 (control group), AUC0 was 0.7, AUC1 was 0.8, Lower false positive rate (FPR) = 0.00, Upper FPR = 1.00. The results indicated that 143 subjects were required for each group. The receiver operating characteristic (ROC) curve was used to represent the prediction model for EV hemorrhage, with the area under the curve (AUC) indicating the value of the prediction model. AUC is an effective way to summarize the overall diagnostic accuracy of the test. It takes values from 0 to 1 (100%), where a value of 0 indicates an inaccurate test and a value of 1 (100%) reflects a perfectly accurate test. Sensitivity, in this case, was the ability to detect true EVH+ patients based on one or more indicators, while specificity was the ability to identify patients with EVs that did not experience bleeding, based on a specific index. The best cutoff value was determined using Youden's index, which is the sum of sensitivity and specificity minus 1. It is the ability to distinguish between EVH+ patients and EVH- patients. The higher the Youden's index (1 or 100%), the more valid the correlation.

## Results

### Patient Characteristics

Baseline patient demographic and clinical characteristics are presented in Table 1. Patient age range was 27–74 years, median age was 52.5 years, and 31.1% (89/286 were female (Table 1). The etiologies of cirrhosis included hepatitis B, hepatitis C, alcoholic liver disease, autoimmune hepatitis, Budd-Chiari syndrome, and drug-induced liver injury (Table 1). There were 71 cases of Child-Pugh class A cirrhosis (well-compensated disease, good hepatic function), 159 cases of class B (significant functional compromise, moderately impaired hepatic function) and 56 cases of class C cirrhosis (decompensated disease, advanced hepatic dysfunction). 39 patients had diabetes mellitus, 128 patients (36 female) had EVs with hemorrhage (EVH+), and 158 patients (53 female) had EVs without hemorrhage (EVH-) (Table 2). The median age was 53 years for EVH+ patients and 52 years in the EVH- group, however this difference was not statistically significant (Table 2).

**Table 1:** Baseline patient characteristics.

**Characteristic**	**Patient # (n)**	**Median (IQR)**
Age (years)	286	52.5 (48.0–58.0)
Sex (M/F)	197/89	n/a
ALB (g/L)	286	33.6 (30.2–37.5)
ALT (U/L)	286	24.0 (17.0–34.0)
AST (U/L)	286	30.0 (23.0–40.0)
TBIL (μmmol/L)	286	19.7 (13.7–32.2)
PT (second)	284	13.9 (12.8–15.4)
APRI	286	1.4 (1.0–2.1)
FIB−4	286	6.1 (4.0–8.7)
GP73 (ng/mL)	148	132.3 (98.3–205.2)
C–P (A/B/C)	71/159/56	n/a
Length of spleen (mm)	279	166.0 (147.0–186.0)
Diabetes (Y/N)	39/247	n/a
Hemorrhage (Y/N)	128/158	n/a
Pathogenesis	287	n/a
HBV	213	n/a
HCV	17	n/a
Alcohol	21	n/a
Other	36	n/a

*ALB, Albumin; ALT, alanine aminotransferase; AST, aspartate aminotransferase; TBIL, total bilirubin; PT, prothrombin time; APRI, AST to PLT ratio index; FIB-4, fibrosis-4; GP73, Golgi protein 73, C-P, Child-Pugh; A/B/C, score. Values are expressed as medians with interquartile range (IQR, 25–75%)*.

**Table 2:** Comparisons between patients with and without esophageal variceal hemorrhage.

**Characteristic**	**Hemorrhage**	**(+)**	**Hemorrhage**	**(–)**	
	**(n)**	**Median (IQR)**	**(n)**	**Median (IQR)**	***P* value**
Age (years)	128	53.0 (47.3–58.0)	158	52.0 (48.0–58.0)	0.776
Sex (M/F)	92/36	n/a	105/53	n/a	0.325
ALB (g/L)	128	31.2 (27.8–34.5)	158	35.7 (32.6–38.3)	*** <0.001
ALT (U/L)	128	24.0 (16.3–34.0)	158	24.0 (17.0–33.3)	0.94
AST (U/L)	128	31.0 (23.0–41.0)	158	29.0 (23.0–40.0)	0.716
TBIL (μmmol/L)	128	20.9 (13.9–38.3)	158	18.6 (13.5–27.2)	*0.046
PT (second)	127	14.7 (13.3–16.6)	157	13.5 (12.4–14.6)	*** <0.001
APRI	128	1.5 (1.0–2.4)	158	1.4 (0.9–2.0)	0.229
FIB−4	128	6.2 (4.0–10.0)	158	6.0 (3.9–8.4)	0.215
GP73 (ng/mL)	63	157.2 (106.9–252.8)	85	120.4 (92.32–171.2)	*0.012
PLT (10^9^ cell/L)	128	56.5 (39.3–70.5)	158	50.4 (40.0–81.0)	0.409
(C-P) (A/B/C)	10/79/39	n/a	61/80/17	n/a	*** <0.001
Length of spleen (mm)	124	164.0 (145.0–186.0)	154	167.0 (150.0–187.0)	0.753
Diabetes (Y/N)	18/110	n/a	21/137	n/a	0.85

ALB, Albumin; ALT, alanine aminotransferase; AST, aspartate aminotransferase; TBIL, total bilirubin; PT, prothrombin time; APRI, AST to PLT ratio index; FIB-4, fibrosis-4; GP73, Golgi protein 73; PLT, platelet count; C-P, Child-Pugh; A/B/C, score. Values are expressed as medians with interquartile range (IQR, 25–75%). *P < 0.05, **P < 0.01, ***P < 0.001.

### Comparisons Between Patients With and Without Esophageal Variceal Hemorrhage

There were no significant differences between the EVH+ and EVH- groups in median values for ALT (24.0 vs. 24.0 U/L, *P* = 0.940), AST (31.0 vs. 29.0 U/L, *P*=0.716), PLT (56.5 × 10^9^ vs. 50.4 × 10^9^/L, *P* = 0.409), spleen length (164 vs. 167 mm, *p* = 0.753), APRI (1.5 vs. 1.4, *P* = 0.229) or FIB-4 (6.2 vs. 6.0, *P* = 0.215) (Table 2). However, the median serum albumin level was significantly lower in the EVH+ group than in the EVH- group (31.2 vs. 35.7 g/L, *P* < 0.0001) (Table 2). Furthermore, median TBIL (20.9 vs. 18.6 μmol/L, *P* < 0.046), PT time (14.7 vs. 13.5 second, *P* < 0.001), and GP73 (157.2 vs. 120.4 ng/mL, *P* = 0.012) were significantly higher in EVH+ patients than in EVH- patients (Table 2). The Child-Pugh class (A, B, and C) also significantly differed between the EVH+ and EVH- groups (10, 79, and 39 among EVH+ patients vs. 61, 80, and 17 among EVH- patients, *P* < 0.001).

### Diagnostic Accuracy of Non-invasive Tests for Predicting EV Hemorrhage

**Figure 1:**
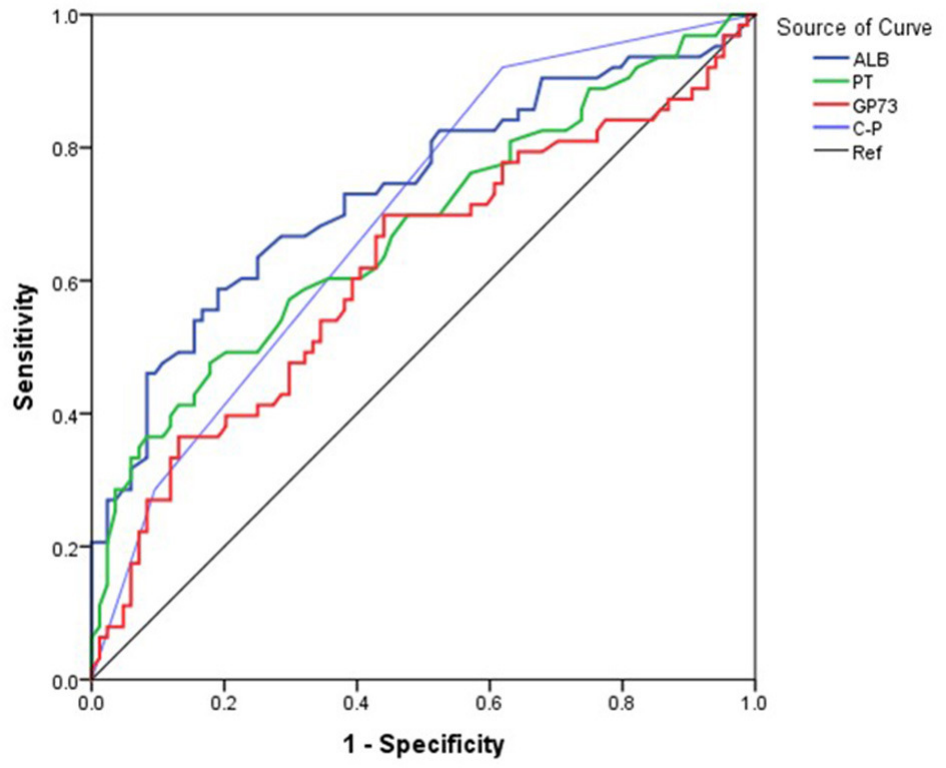
Diagnostic accuracy of individual variables in the prediction of esophageal variceal hemorrhage. The ROC curve was used to represent the prediction model for hemorrhage using a single clinical marker, with the AUC indicating the value of the prediction model. The ROC curve was plotted based on the data in Table 3. Different colored lines represent different predictors. Blue: ALB, Green: PT, Red: GP73, Purple: (C-P), Black: Reference (Ref).

**Figure 2:**
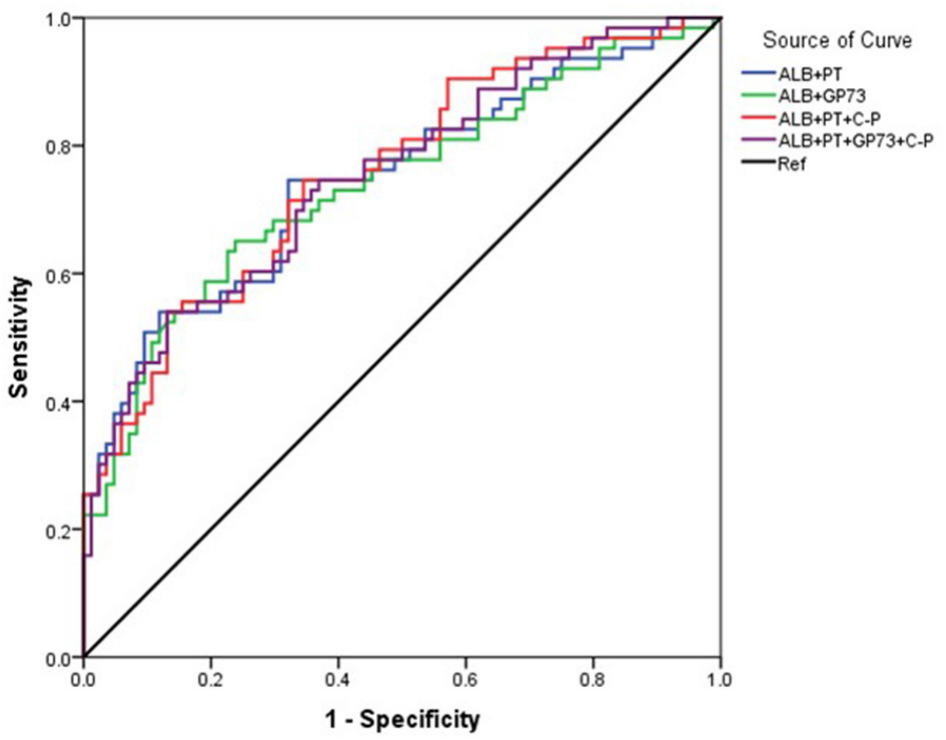
Diagnostic accuracy of parallel combination of variables for the prediction of esophageal variceal hemorrhage. The ROC curve was used to represent the prediction model for hemorrhage using multiple clinical markers, with the AUC indicating the value of the prediction model. Different colors represent different predictors. The ROC curve was plotted based on the data in Table 4. Blue: ALB+PT, Green: ALB+GP73, Red: ALB+PT+(C-P), Purple: ALB+PT+GP73+ (C-P), Black: Reference (Ref).

The area under the curve (AUC) for ALB was 73.4% (95% CI 67.5–79.2), with a cutoff of 33.2 g/L, sensitivity of 71.5%, and specificity of 65.6%. The AUC for PT was 68.6% (95% CI 62.4–74.8) with a cutoff 14.2 seconds, sensitivity of 59.8%, and specificity of 70.1%. The AUC (95% CI 52.8–71.5) for GP73 was 62.2% with a cutoff 126.4 ng/ml, sensitivity of 69.8%, and specificity of 56.5% (Figure 1, Table 3). The AUC for Child-Pugh (C-P) scores was 69.8% (95%CI 63.8–75.8) with a sensitivity of 92.2% and specificity of 38.6% (Figure 1, Table 3). We next combined several indicators to improve the accuracy of predictions. The AUC for the combination of ALB and GP73 in predicting EV hemorrhage was 74.3% (95% CI 66.1–82.5) with a sensitivity of 65.1% and specificity of 76.5% (Figure 2, Table 4). Parallel combination of ALB, PT, and C-P scores had an AUC of 76.5% (95% CI 70.9–82.1) with a sensitivity of 79.5% and specificity of 64.3% (Figure 2, Table 4). When ALB, PT, GP73, and C-P scores were combined the AUC was 75.2% (95% CI 67.3–83.1), with a sensitivity of 54.0% and specificity of 86.9% (Figure 2, Table 4). Overall, the diagnostic accuracy of multiple non-invasive clinical markers combined in parallel was higher than that obtained by each individual marker (Tables 3, 4).

**Table 3:** Diagnostic accuracy of single variables in predicting esophageal variceal hemorrhage.

**Characteristic**	**AUC% (95% CI)**	**Cutoff**	**Sensitivity**	**Specificity**	**Youden's**
		**(%)**	**(%)**	**(%)**	**index (%)**
ALB (g/L)	73.4(67.5–79.2)	33.2	71.5	65.6	37.1
PT (second)	68.6(62.4–74.8)	14.2	59.8	70.1	29.9
GP73 (ng/mL)	62.2(52.8–71.5)	126.4	69.8	56.5	26.3
(C-P)	69.8(63.8–75.8)	-	92.2	38.6	30.8

*ROC, Receiver-operating characteristic; AUC, area under the curve; ALB, albumin; PT, prothrombin time; GP73, Golgi protein 73; PLT, platelet count; C-P score, Child-Pugh. Values are expressed as medians with interquartile range (IQR, 25–75%)*.

**Table 4:** Diagnostic accuracy of parallel combination of variables in the prediction of esophageal variceal hemorrhage.

**Characteristic**	**AUC% (95% CI)**	**Cutoff**	**Sensitivity**	**Specificity**	**Youden's**
		**(%)**	**(%)**	**(%)**	**index (%)**
ALB+PT	75.9(70.2–81.6)	40.5	78.7	66.9	45.6
ALB+GP73	74.3(66.1–82.5)	44.3	65.1	76.5	41.6
ALB+PT+ (C-P)	76.5(70.9–82.1)	40.6	79.5	64.3	43.8
ALB+PT+ GP73+(C-P)	75.2(67.3–83.1)	53.0	54	86.9	40.9

*ROC, receiver-operating characteristic; AUC, area under the curve; ALB, albumin; PT, prothrombin time; GP73, Golgi protein 73; PLT, platelet count; C-P, Child-Pugh; score. Values are expressed as medians with interquartile range (IQR, 25–75%)*.

## Discussion

Non-invasive tests that can accurately predict bleeding from EVs in cirrhosis may allow certain patients to avoid invasive screening upper endoscopy. Several non-invasive methods that identify EVs in patients with compensated cirrhosis have already been proposed. For example, it has been reported that acoustic radiation force impulse (ARFI) elastography can accurately diagnose high-risk EVs (28). It has also been shown that liver and spleen stiffness measurements correspond with the hepatic venous pressure gradient (HVPG) and could thus be used to diagnose portal hypertension (8). The Baveno IV consensus recommended that patients with a platelet count >150,000 and LS measurement <20 kPa do not require EV screening by EGD (12). However, if capacity for TE or EGD is limited or patients that do not meet these conditions wish to avoid upper GI endoscopy, other simple, readily available biomarkers may allow cirrhotic patients to be stratified based on their risk for EV hemorrhage or which patients may benefit from prophylactic NSBB therapy. Furthermore, although TE and platelet count may have clear clinical value for predicting EVH, there are deviations in patients with ascites and abnormal liver function. The aim of this study, therefore, was to analyze a combination of simple, readily available biomarkers for the prediction of EV hemorrhage.

We found that ALB, PT, GP73 and C-P scores were all associated with hemorrhage in cirrhotic patients with EVs. Moreover, EVH+ patients had lower ALB, higher TBIL, PT, GP73 and C-P scores compared to EVH- patients. As an individual marker, ALB (cutoff of 33.2 g/L) had the highest AUC% with a high degree of sensitivity and specificity. The sensitivity of C-P scores in predicting EV hemorrhage was 92.2% but the specificity was low (38.6%). Therefore, the C-P score has more value as a screening test to rule out high-risk varices among patients with cirrhosis. Since the application of a single clinical marker to predict EV hemorrhage had limitations in terms of accuracy, sensitivity, and specificity, we then sought to determine whether a combination of several different clinical markers could improve diagnostic accuracy. We found that ALB combined with PT or GP73 improved accuracy, sensitivity, and specificity. The combinations of ALB, PT and C-P or ALB, PT, GP73, and C-P slightly increased the AUC%. Parallel combination of ALB, PT, GP73, and C-P increased the specificity to 86.9% although this resulted in low sensitivity (54%). The combination of ALB, TBIL, GP73, and C-P scores as a single index may thus be developed as a composite score for the prediction of variceal hemorrhage and further study is warranted.

There are a few limitations to this retrospective study. Notably, the accuracy of NITs in identifying patients with high- or low-risk varices was not assessed, as the patient cohort overall had similarly graded EVs. We therefore explored which factors are associated with EV hemorrhage. In addition, the sample size was relatively small and GP73 was not measured in all patients. A longer evaluation period and larger sample size would therefore permit the identification of markers that can discriminate between high- and low-risk varices.

## Data Availability Statement

The raw data supporting the conclusions of this article will be made available by the authors, without undue reservation.

## Ethics Statement

This study was registered at the Chinese Clinical Trial Registry (Registration Number: ChiCTR2000034735). The study protocol was approved by the Ethics Review Committee of the Second People's Hospital of Fuyang City (Reference Number: 2019006). This research was conducted in accordance with the Ethical Standards of the Institutional and National Research Committees, and with the 1964 declaration of Helsinki. The patients/participants provided their written informed consent to participate in this study.

## Author Contributions

SL, PH, AJ, MH, LT, and WL designed the research study, analyzed and interpreted the data, and wrote the manuscript. SL, CM, KZ, CZ, NJ, ML, TS, MH, and LT were involved in diagnosis and treatment of patients, recruiting patients and collection of clinical data. All authors were involved in critical appraisal of the manuscript and approved the final version of the manuscript.

## Funding

This study was funded in part by grant of Natural Science Foundation of China (NSFC 81871661 to WL), the Research Grant of Fuyang City Department of Science and Technology (FK202081051 to SL).

## Conflict of Interest

The authors declare that the research was conducted in the absence of any commercial or financial relationships that could be construed as a potential conflict of interest.

## Publisher's Note

All claims expressed in this article are solely those of the authors and do not necessarily represent those of their affiliated organizations, or those of the publisher, the editors and the reviewers. Any product that may be evaluated in this article, or claim that may be made by its manufacturer, is not guaranteed or endorsed by the publisher.
